# Dectin-2 mediates phagocytosis of *Lactobacillus paracasei* KW3110 and IL-10 production by macrophages

**DOI:** 10.1038/s41598-021-97087-9

**Published:** 2021-09-06

**Authors:** Mia Yoshikawa, Sayuri Yamada, Miho Sugamata, Osamu Kanauchi, Yuji Morita

**Affiliations:** KIRIN Central Research Institute, Kirin Holdings Company, Ltd., Tokyo, Japan

**Keywords:** Biotechnology, Microbiology, Molecular biology

## Abstract

Lactic acid bacteria (LAB) are most generally used as probiotics and some strains of LAB are known to have anti-inflammatory effects. A specific strain of lactic acid bacteria, *Lactobacillus paracasei* KW3110 (KW3110), activates macrophages to produce interleukin-10 (IL-10), an anti-inflammatory cytokine; however, the biological mechanism remains unclear. In this study, we showed that the amount of incorporated KW3110 into a macrophage cell line, RAW 264.7, was higher than other genetically related strains using fluorescence microscopy. RNA-seq analysis indicated that treatment of macrophages with KW3110 induced Dectin-2 gene expression, which is a pattern recognition receptor, recognizing α-mannose. In addition, antibody treatment and knock down of Dectin-2, or factors downstream in the signaling pathway, decreased the amount of incorporated KW3110 and IL-10 production. Substantial lectin array analysis also revealed that KW3110 had higher binding affinities to lectins, which recognize the carbohydrate chains comprised of α-mannose, than two other LAB. In conclusion, KW3110 is readily incorporated into macrophages, leading to IL-10 production. Dectin-2 mediated the phagocytosis of KW3110 into macrophages and this may be involved with the characteristic carbohydrate chains of KW3110.

## Introduction

Lactic acid bacteria (LAB) are widely known to have suppressive effects on inflammatory diseases through the induction of anti-inflammatory cytokines, such as IL-10. We previously demonstrated that *L. paracasei* KW3110, a specific strain of LAB, significantly induced IL-10 production in macrophages through nucleotide oligomerization domain (NOD) 2 signaling pathway^1^. Many studies have suggested that the Toll-like receptor (TLR) 2 pathway is involved in IL-10 induction by LAB in macrophages; however, our experiments showed that blocking TLR2 using neutralizing antibodies does not affect IL-10 production by KW3110 stimulation. Our results suggest that TLR2 is not crucial for IL-10 production induced by KW3110^2^. Although we have shown that KW3110 has strong anti-inflammatory effects^3^, the molecular mechanism underlying increased IL-10 production remains unclear.

Macrophages play a significant part in the immune response to injury by engulfing and digesting particles, including pathogens or bacteria, in a process called phagocytosis. It is known that macrophages produce IL-10 during phagocytosis of apoptotic cells^4^. During the first step of phagocytosis, the pattern-recognition receptors (PRRs) on the surface of macrophages, such as TLRs and C-type lectin receptors (CLRs), adhere to the surface of microbes or other particles. Among these receptors, CLRs are frequently reported to have an essential role in taking up microbes or other particles into phagocytic cells^5,6^; therefore, CLRs are often called endocytic/phagocytic receptors or antigen-uptake receptors. Many types of CLRs, such as Dectin-1, Dectin-2 and Mincle, have been proposed to individually participate in the immune response to microbes, through binding to carbohydrates. While Dectin-1 recognizes β-glucans, Dectin-2 and Mincle recognize mannosylated ligands^7^; however, it is not known whether recognition of KW3110 by macrophages is related to these CLRs. Intracellular signaling pathways involved in phagocytosis are also important in inducing IL-10 production. After phagocytosis, the fractions which are engulfed and internalized by endocytosis are collectively digested or escape from the endosome via endosomal peptide transporters. Proton coupled peptide transporter (POT), a member of a major transporter family, are expressed in all organisms and use the proton electrochemical gradient to drive the uptake of peptides across cell membranes^8,9^. POTs are responsible for the egress of fragments containing muramyl dipeptides (MDP). It is known that bacterial MDP stimulated a type of POT in RAW 264.7 cells which, in turn, facilitated the NOD2-dependent immune response^10^. However, there are no reports that they contribute to the production of IL-10 by macrophages through phagocytosis.

Carbohydrates within the microbial cell wall are known to trigger reorganization and phagocytosis by macrophages. In a recent study using lectin array, a tool to investigate carbohydrates, it was shown that the patterns of lectin-binding affinity differ between the strains of LAB^11^. The results indicated that LAB have different types of cell surface carbohydrates and the difference leads to a variety of immune responses via differential mechanisms. There are very few reports on cell-microbe interactions via bacterial carbohydrate chains; therefore, it remains unclear whether characteristic cell surface carbohydrates within strains are involved in phagocytosis.

In the present study, we investigated the involvement of phagocytosis in increased IL-10 production in RAW 264.7 cells, a macrophage-like cell line. We examined the role of CLRs on the surface of RAW 264.7 cells as a binding target for KW3110 using RNA-seq, siRNA and antibody treatment. We also performed lectin array analysis to clarify the characteristic carbohydrate chains of KW3110.

## Results

### KW3110 induces IL-10 production in RAW 264.7 cells

**Figure 1:**
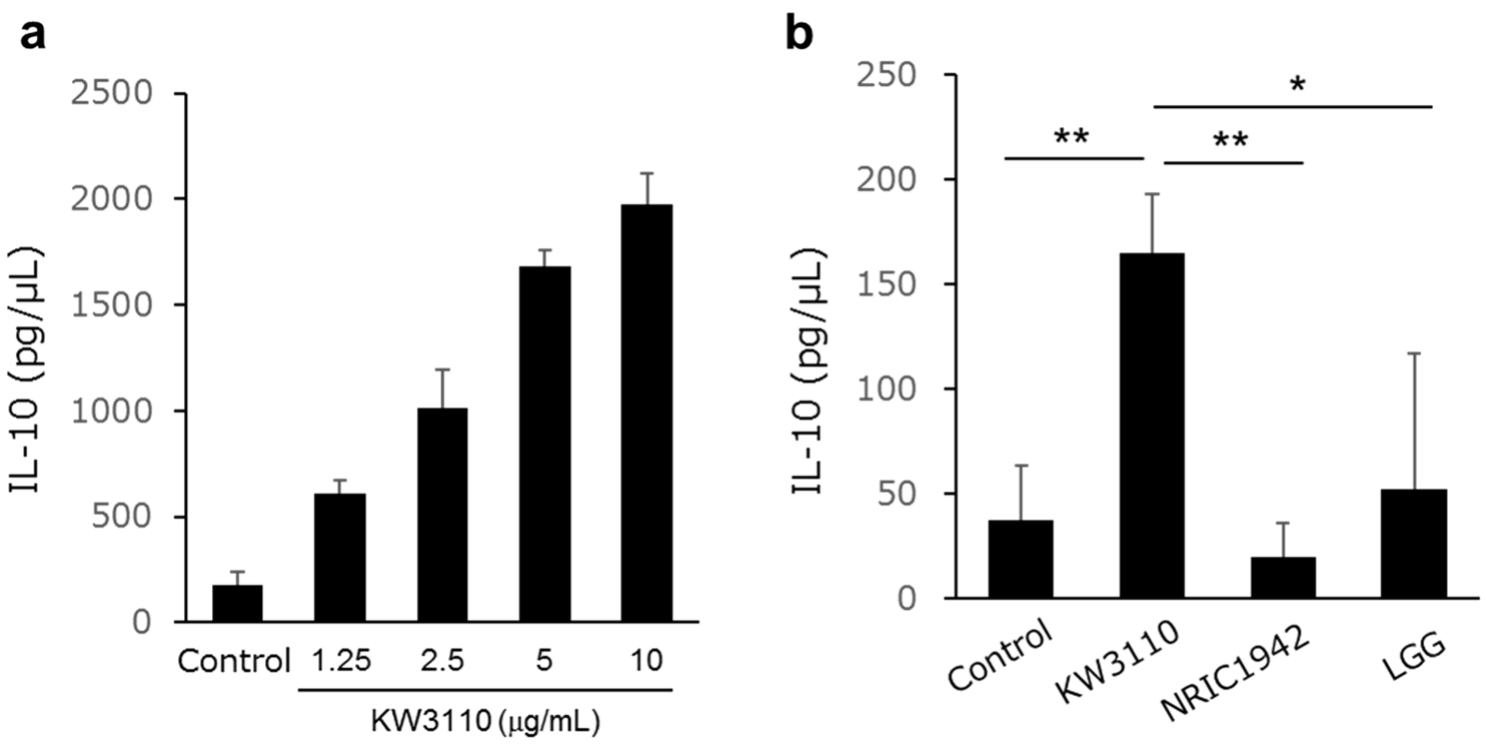
Treatment with KW3110 induces IL-10 secretion. (**a**) RAW 264.7 cells were treated with different doses of KW3110 for 24 h. IL-10 concentration in the supernatants was measured by ELISA. (**b**) RAW 264.7 cells were treated with KW3110, NRIC1942, and LGG for 24 h. IL-10 concentration in the supernatants was measured by ELISA. Significant differences were compared to the control group, *p < 0.05, **p < 0.01. Data are the means ± SD. Data are representative of at least three independent experiments.

RAW 264.7 cells were stimulated with KW3110, *L. paracasei* NRIC1942, or *L. rhamnosus* GG (LGG) for 24 h. KW3110 and NRIC1942 are strains of same species. LGG is one of the most well-studied LAB with regard to its effect on human health^12^, and is known to induce IL-10 in macrophages^13^. We demonstrated that IL-10 production was increased in RAW 264.7 cells treated with KW3110 in a dose-dependent manner (Fig. 1a). KW3110 significantly induced much higher levels of IL-10 production in RAW 264.7 cells than NRIC1942 or LGG (Fig. 1b).

### KW3110 is efficiently incorporated into RAW 264.7 cells

The phagocytosis of several ligands, including bacteria, into RAW 264.7 cells activated IL-10 production^14^; therefore, we evaluated whether KW3110, LGG and NRIC1942 are incorporated into RAW 264.7 cells and induce IL-10 production.

**Figure 2:**
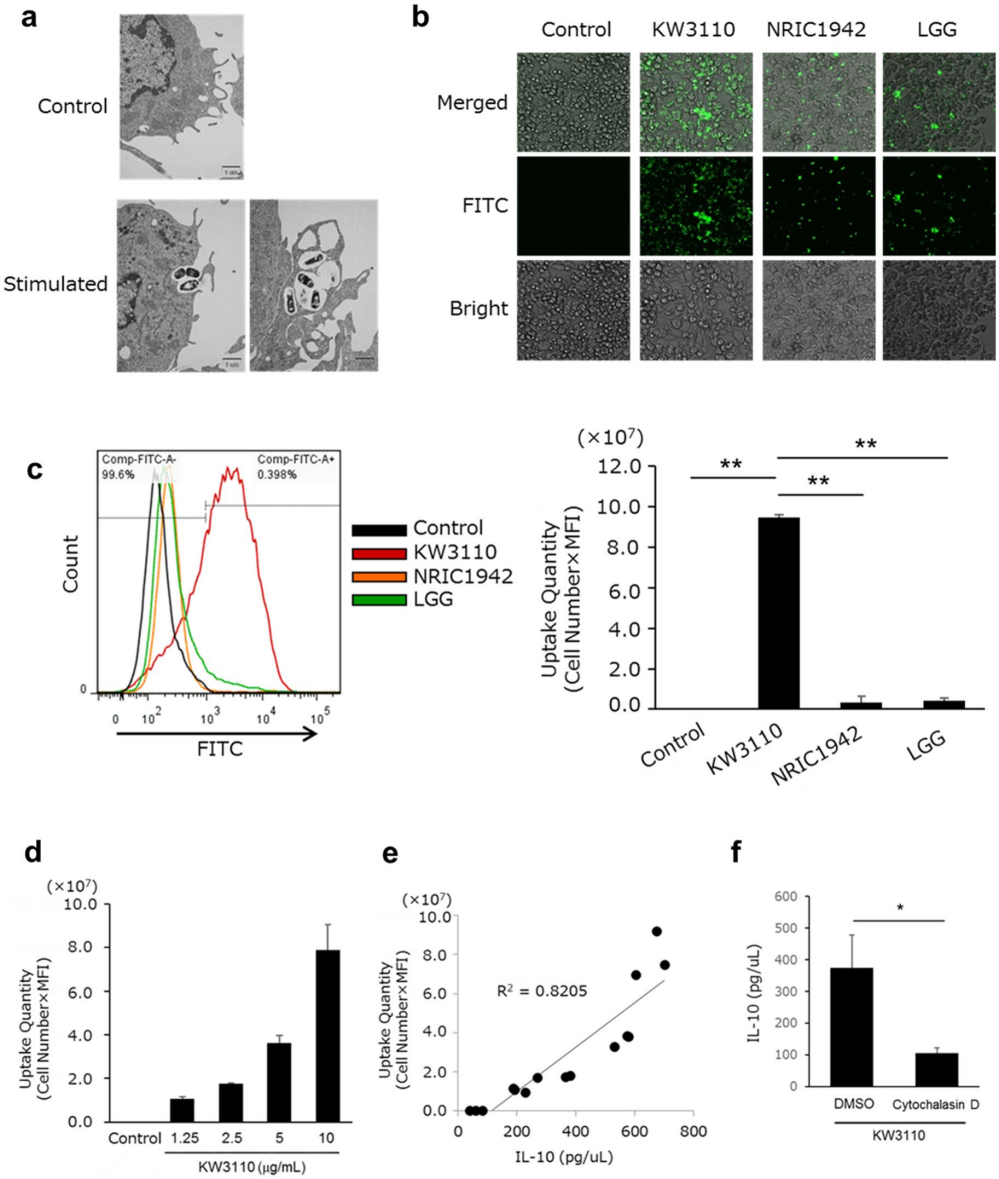
KW3110 is incorporated into RAW 264.7 cells. (**a**) RAW 264.7 cells were incubated with KW3110 for 2–4 h. The incorporation was visualized by TEM. (**b**) RAW 264.7 cells were incubated with FITC-labeled *Lactobacilli* for 24 h. The cells were observed by confocal laser scanning microscopy. (**c**) RAW 264.7 cells were incubated with FITC-labeled *Lactobacilli* for 24 h. The amount of incorporated *Lactobacilli* was measured by flow cytometry. The signal intensity of FITC (*x* axis) versus the number of cells (*y* axis) is shown. Control, KW3110, NRIC1942 and LGG are indicated by back, red, orange and green lines, respectively. Uptake quantities were quantified for statistical analysis. (**d**) RAW cells were treated with different doses of KW3110 for 24 h. The amount of incorporated KW3110 was measured by flow cytometry. (**e**) RAW cells were treated with different doses of KW3110 for 24 h. Correlation between IL-10 production and uptake quantity was calculated. (**f**) RAW cells were treated with KW3110 in the presence of DMSO or cytochalasin D for 24 h. IL-10 concentration in the supernatants was measured by ELISA. Significant differences were compared to the control group, *p < 0.05, **p < 0.01. Data are the means ± SD. Data are representative of at least three independent experiments.

We analyzed KW3110-stimulated RAW 264.7 cells by Transmission electron microscopy (TEM). As shown in Fig. 2a, KW3110 were incorporated into RAW 264.7 cells at least 2 h after stimulation. We also evaluated the incorporation of FITC-labeled LAB into RAW 264.7 cells using fluorescence microscopy and flow cytometry (Fig. 2b,c). Microscopy analysis showed that the amount of KW3110 internalized into RAW 264.7 cells was greater than that of NRIC1942 and LGG internalization (Fig. 2b). In addition, flow cytometry analysis showed that the fluorescence intensity of FITC-labelled KW3110 was higher than the other LAB in RAW 264.7 cells (Fig. 2c). These results indicated that KW3110 was incorporated more efficiently into RAW 264.7 cells than NRIC1942 and LGG. Incorporation into RAW 264.7 cells occurred in a KW3110 dose-dependent manner (Fig. 2d). In addition, a strong correlation was found between the amount of incorporation and IL-10 production (Fig. 2e). On the other hand, IL-10 production in RAW 264.7 cells stimulated by KW3110 was suppressed by a phagocytosis inhibitor (Fig. 2f).

### *Cleac4n*, gene symbol of Dectin-2, was up-regulated in response to KW3110

**Figure 3:**
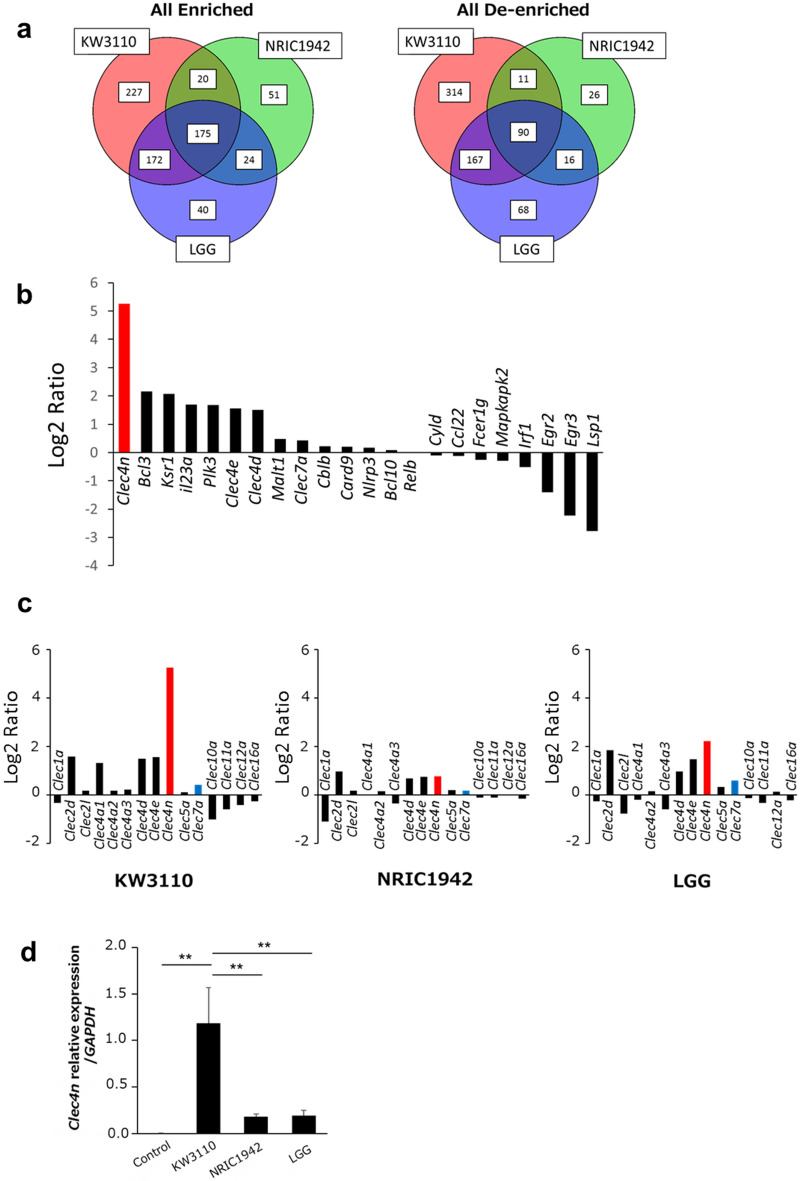
Dectin-2 involvement in KW3110 phagocytosis. (**a**) Venn diagram of up- (left) or down- (right) regulated genes in stimulated and non-stimulated RAW 264.7 cells by each *Lactobacilli*. (**b**) Variance of gene expression in the cluster of C-type lectin receptor genes after stimulation by KW3110. The cluster is defined by KEGG. The gene expressions were compared between KW3110 stimulated and non-stimulated RAW 264.7 cells. *Clec4n* gene expression is represented by a red bar. (**c**) Gene expression variance of C-type lectin domain genes when stimulated by each *Lactobacilli*. Gene expressions were compared between stimulated and non-stimulated RAW 264.7 cells. The gene expression of *Clec4n* and *Clec7a* are represented by red and blue bars. (**d**) *Clec4n* expression in RAW cells stimulated by *Lactobacilli* (relative expression levels normalized to GAPDH). Significant differences were compared to the control group, **p < 0.01. Data are the means ± SD. Data are representative of at least three independent experiments.

To identify the pathways potentially involved in phagocytosis of KW3110 by RAW 264.7 cells, we performed RNA-seq analysis to investigate gene expression in RAW 264.7 cells treated with KW3110, NRIC1942, or LGG. Although many genes are up- or down- regulated in response to LAB species stimulation (Fig. 3a), gene expression was more altered in KW3110-stimulated RAW 264.7 cells when compared with cells stimulated with the other two species. Among the genes whose expression level changed, we focused on CLRs.

The category classification of genes, based on KEGG (Kyoto Encyclopedia of Genes and Genomes), showed that many C-type lectin receptor signaling pathway genes were up- or down-regulated when RAW 264.7 cells were stimulated by KW3110 (Fig. 3b). CLRs are known to recognize microbial cell walls and influence the innate immune system through phagocytosis^15^. *Clec4n* (C-type lectin domain family 4, member N), gene symbol of Dectin-2, showed the highest amount of up-regulation by KW3110 compared with NRIC1942 and LGG. *Clec7a* (C-type lectin domain family 7, member A), gene symbol of Dectin-1, which belongs to the same protein family as Dectin-2, was not up-regulated by KW3110 (Fig. 3c). *Clec4n* was up-regulated in RAW 264.7 cells after stimulation by KW3110 and LGG but not NRIC1942. Upregulation of *Clec4n* was much higher in KW3110-stimulated RAW 264.7 cells than LGG-stimulated cells.

To confirm the gene expression levels obtained in the RNA-seq analysis, expression of *Clec4n* was evaluated using real-time PCR (Fig. 3d). The mRNA expression levels of *Clec4n* were significantly up-regulated only when stimulated by KW3110: the mRNA expression levels were not changed when stimulated by NRIC1942 or LGG.

### Dectin-2 involvement in KW3110 phagocytosis

**Figure 4:**
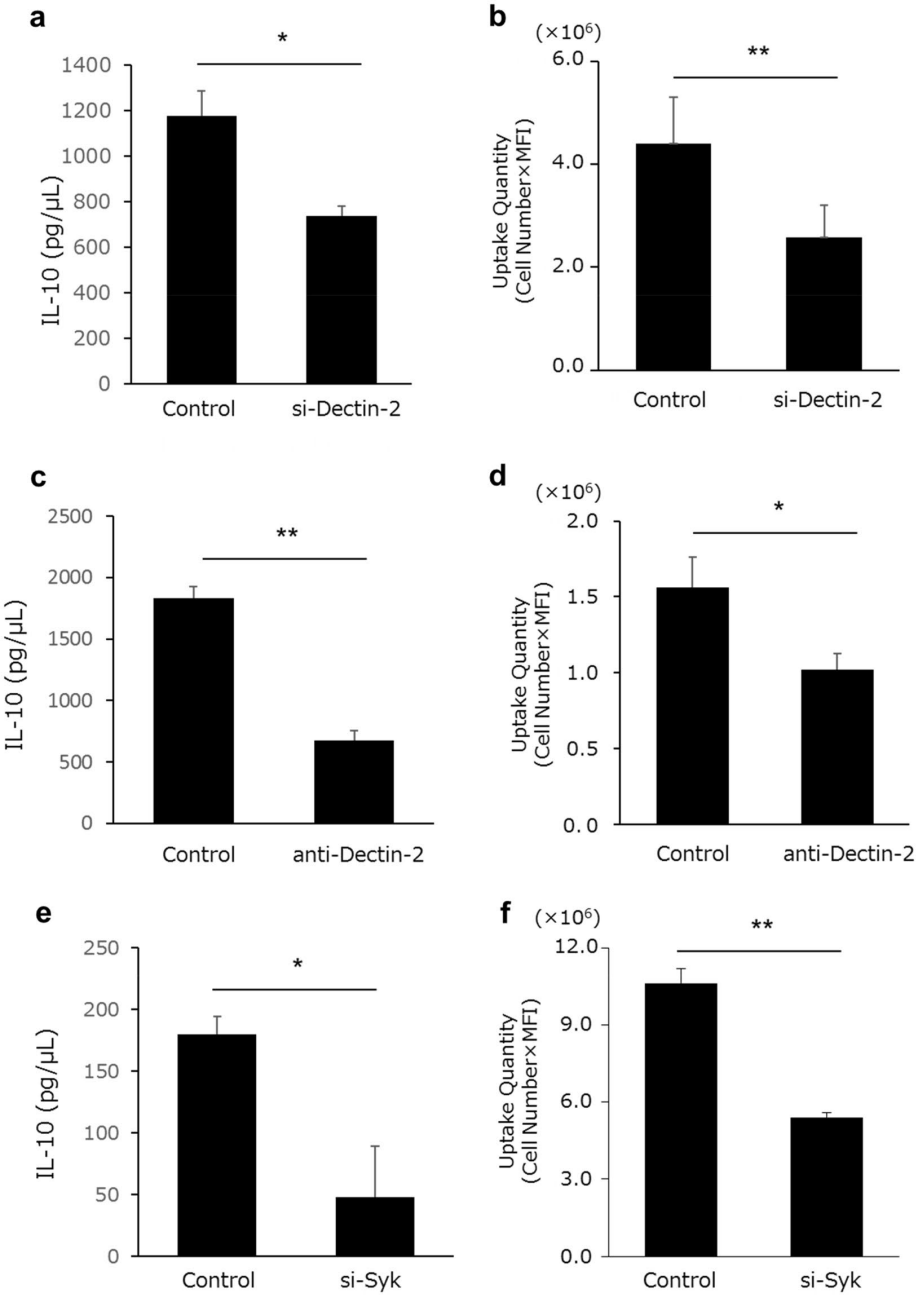
Inhibition of Dectin-2-syk pathway abolishes the effect of KW3110 on IL-10 production. (**a**, **b**) Dectin-2 knockout RAW 264.7 cells were treated with KW3110 for 24 h. (**a**) IL-10 concentration in the supernatants was measured by ELISA. (**b**) The amount of incorporated KW was measured by flow cytometry. (**c**, **d**) RAW 264.7 cells were treated with KW3110 in the presence or absence of anti-Dectin-2 antibody for 24 h. (**c**) IL-10 concentration in the supernatants was measured by ELISA. (**d**) The amount of incorporated KW was measured by flow cytometry. (**e**, **f**) Syk knockout RAW 264.7 cells were treated with KW3110 for 24 h. (**e**) IL-10 concentration in the supernatants was measured by ELISA. (**f**) The amount of incorporated KW was measured by flow cytometry Significant differences were compared to the control group, *p < 0.05, **p < 0.01. Data are the means ± SD. Data are representative of at least three independent experiments.

To clarify the role of Dectin-2 in the phagocytosis of KW3110 into RAW 264.7 cells, we conducted siRNA and ELISA analyses. Knockdown of Dectin-2 (si-Dectin-2) significantly suppressed the incorporation of KW3110 into RAW 264.7 cells and IL-10 production when compared with the control siRNA (si-Control) cells (Fig. 4a,b). On the other hand, knockdown of Dectin-1 (si-Dectin-1), which belong to the same family as Dectin-2, did not decrease the incorporation of KW-3110 and IL-10 production (Supplemental Fig. 2a,b). Consistent with the siRNA treatment, pretreatment with anti-Dectin-2 antibody decreased incorporation of KW3110 into RAW 264.7 cells and IL-10 production when compared with the control cells (Fig. 4c,d). Dectin-2 activates the downstream protein-tyrosine kinase, Syk^16^, which plays an essential role in phagocytosis in macrophages^17^. The mRNA expression levels of *Syk* were significantly up-regulated after KW3110 stimulation of RAW 264.7 cells (Supplemental Fig. 2c). We evaluated the amount of KW3110 incorporated and the KW3110-induced IL-10 production from si-Syk RAW 264.7 cells. As expected, both the amount of KW3110-incorporation into si-Syk RAW 264.7 cells and IL-10 production from si-Syk RAW 264.7 were significantly decreased (Fig. 4e,f).

### PHT2 is involved in transport of phagocytosed KW3110

**Figure 5:**
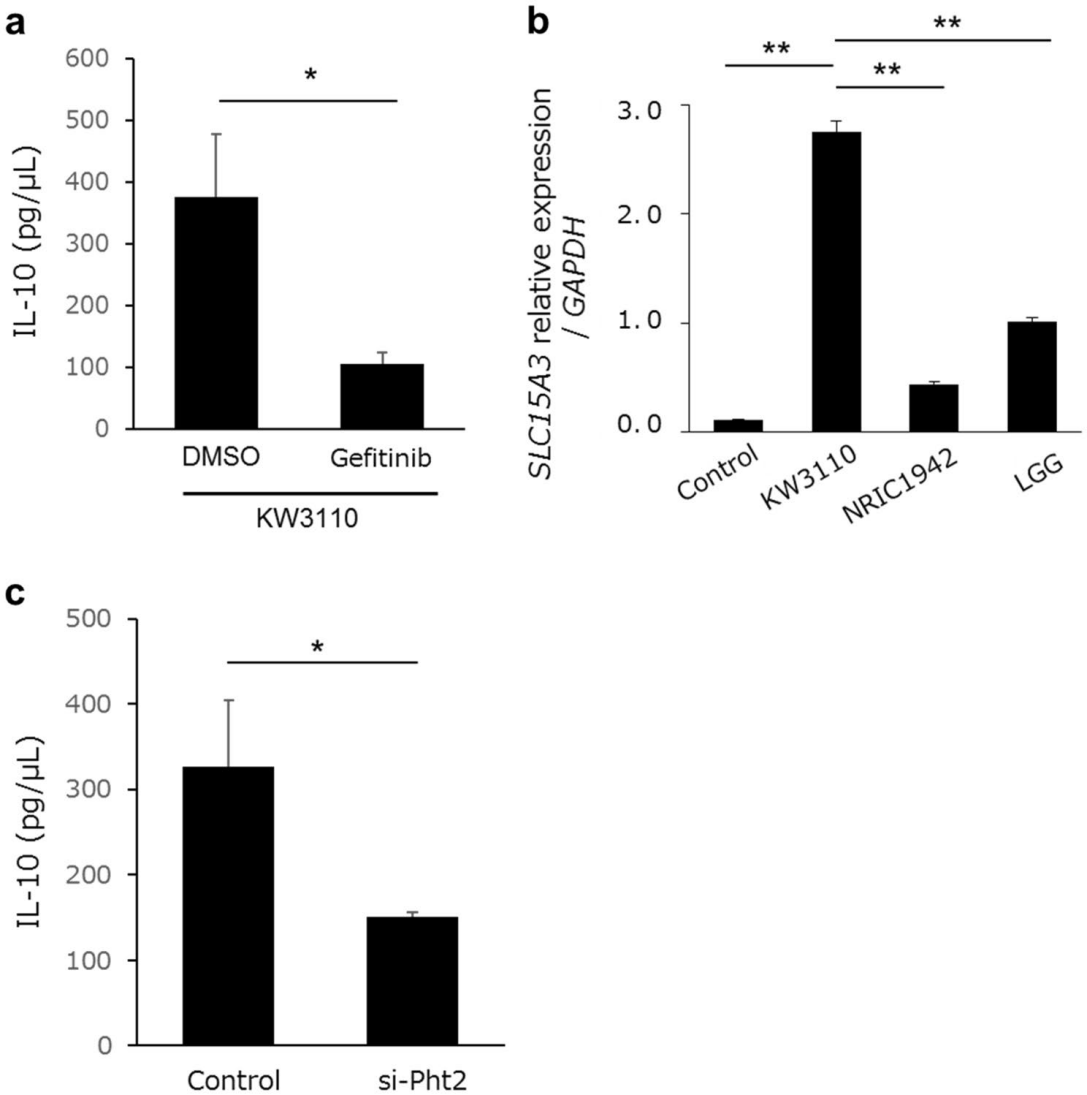
NOD2 signaling and Pht2 transporter are involved in IL-10 production in RAW 264.7 cells stimulated by KW3110. (**a**) RAW 264.7 cells were treated with KW3110 in the presence of DMSO or Gefitinib for 24 h. IL-10 concentration in the supernatants was measured by ELISA. (**b**) *SLC15A3* expression in RAW 264.7 cells stimulated by *Lactobacilli*. Relative gene expression levels were normalized to GAPDH. (**c**) Pht-2 knockout RAW 264.7 cells were treated with KW3110 for 24 h. IL-10 concentration in the supernatants was measured by ELISA. Significant differences were compared to the control group, *p < 0.05, **p < 0.01. Data are the means ± SD. Data are representative of at least three independent experiments.

We evaluated the Dectin-2 downstream signaling pathway in RAW 264.7 cells. It is already known that IL-10 production in macrophages by KW3110 stimulation is dependent on phagocytosis and the NOD2 signaling pathway^2^. Consistent with the previous study, we confirmed that pretreatment with NOD2 signaling inhibitor (Gefitinib) suppressed the effect of KW3110 on IL-10 production in RAW 264.7 cells (Fig. 5a). NOD2 recognizes bacterial peptidoglycan fragments containing muramyl dipeptides (MDP) in the cytoplasm of macrophages^18^. As some members of the POT family are known to be endo-lysosomal peptide transporters^19^, we investigated whether POT family members were involved in the signaling pathway. The POT family is comprised of four protein members: PEPT1 (gene symbol: *SLC15A1*), PEPT2 (gene symbol: *SLC15A2*), PHT1 (gene symbol: *SLC15A4*) and PHT2 (gene symbol: *SLC15A3*). Quantitative PCR revealed that *SLC15A1* and *SLC15A2* were not expressed in RAW 264.7 cells at the mRNA level (data not shown). Both *SLC15A3* and *SLC15A4* were expressed in RAW 264.7 cells. Whilst *SLC15A3* expression levels increased 23.0-fold after KW3110 stimulation (Fig. 5b), stimulation by NRIC1942 or LGG did not affect the expression of *SLC15A3* in RAW 264.7 cells. *SLC15A4* expression increased 1.9-fold when stimulated by NRIC1942, and 1.2-fold when stimulated by KW3110. Moreover, siRNA of *SLC15A3* (si-PHT2) suppressed IL-10 production in KW3110-stimulated RAW 264.7 cells (Fig. 5c). SiRNA of *SLC15A4* (si-PHT1) did not decrease IL-10 production (Supplemental Fig. 3a).

### Lectin microarray showed that α-mannose-binding lectins recognize KW3110

**Figure 6:**
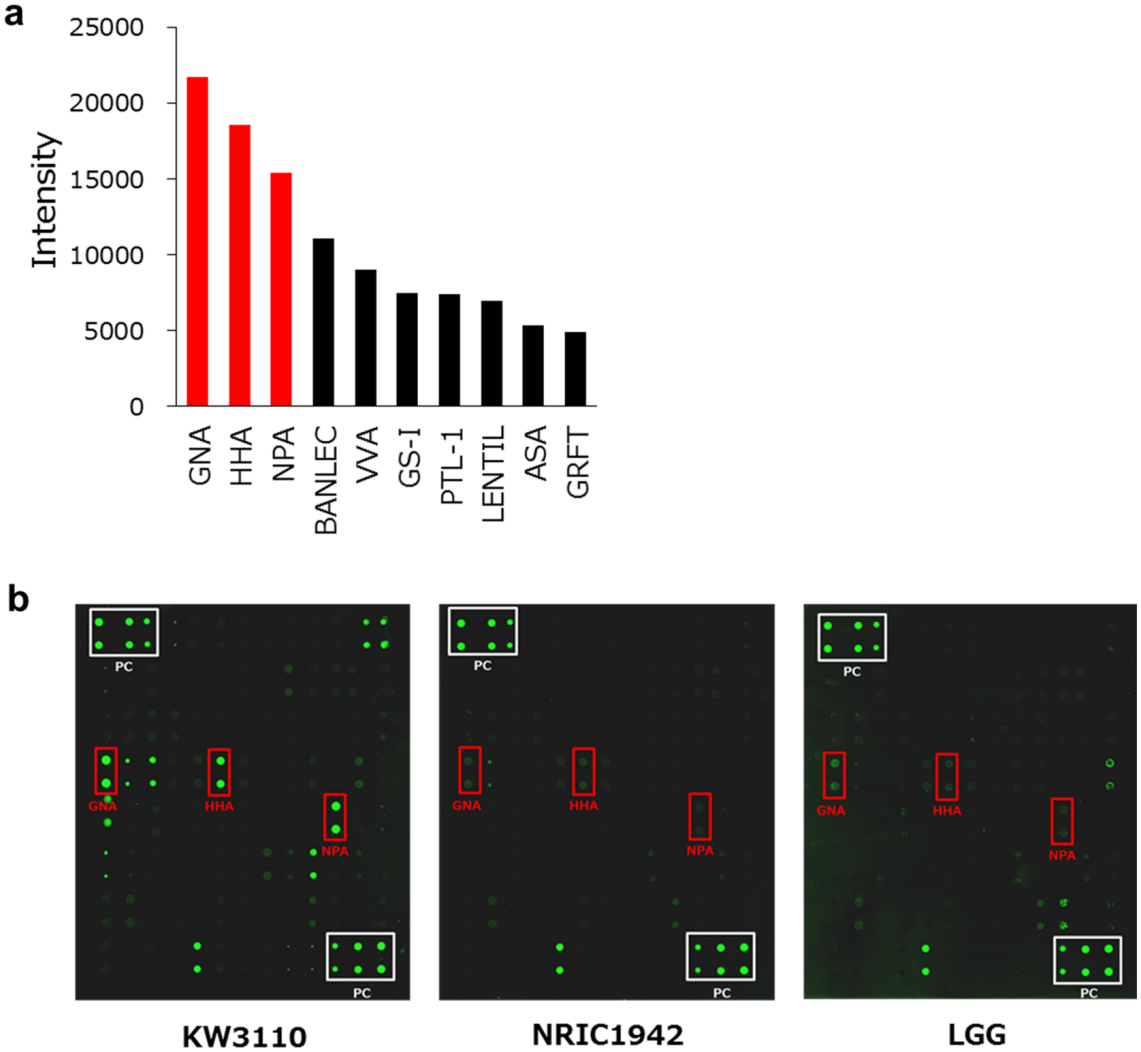
The patterns of lectin-binding affinity differ between the strains of LAB. (**a**) Fluorescence detection images of lectin arrays. Spots enclosed within a white line indicate positive control. The three lectins enclosed within a red line are described in abbreviation, GNA; Galanthus nivalis bulbs, HHA; Hippeastrum hybrid bulbs, NPA; Narcissus pseudonarcissus bulbs. (**b**) The 10 highest intensity lectin array spots after KW3110 addition; top 3 are represented in red. All lectins named are described in abbreviation, *GNA* Galanthus nivalis bulbs, *HHA* Hippeastrum hybrid bulbs, *NPA* Narcissus pseudonarcissus bulbs, *BANLEC* *E. coli* expressed Musa acuminata, *VVA* Vicia villosa seeds, *GS-I* Griffonia simplicifolia seeds, *PTL-1* Psophocarpus tetragonolobus seeds, *LENTIL* Lens culinaris seed, *ASA* Allium sativum agglutinin, *GRFT* *E. coli* expressed Griffithia sp.

To investigate whether the polysaccharides on the surface of KW3110 have high affinity for Dectin-2, we performed lectin microarray analysis. Glycan profiles of KW3110 were mostly occupied by α-mannose-type glycans, including GNA, HHA, and NPA (Fig. 6a,b). The profiles were distinct from those of NRIC1942 and LGG.

## Discussion

LAB are widely known for their anti-inflammatory effects. KW3110 is one of the LAB which is reported to activate macrophages and induce IL-10 production; however, the biological mechanism remains unclear. In this study, we revealed that the phagocytosis process is essential for IL-10 production in macrophages stimulated by KW3110. Dectin-2, which recognizes α-mannose, contributes to phagocytosis of KW3110 in RAW 264.7 cells. Furthermore, lectin microarray experiments revealed that KW3110 had higher binding affinities to lectins, which recognize the carbohydrate chains comprised of α-mannose, than NRIC1942 and LGG. These results suggest that the characteristic carbohydrate chains of KW3110 might contribute to easy incorporation into RAW 264.7 cells.

Pretreatment with cytochalasin D suppressed IL-10 production in KW3110-stimulated RAW 264.7 cells. As cytochalasin D is a phagocytosis blocker, phagocytosis of KW3110 is suggested to be essential for IL-10 production from macrophages. We investigated the carbohydrate chains contained within the cell wall of LAB, which are known determining factors of phagocytosis in macrophages^20^. Carbohydrate chains are inserted into lipid bilayers in cells, as glycoproteins and glycolipids, and are known to be involved in cell–cell or cell-microbe interactions. Some PRRs, such as Mincle and Dectin-2, are known to impact on the clearance of pathogens. PRRs on macrophages interact with the surface carbohydrate of pathogens, leading to promotion of phagocytosis by macrophages^21^. Carbohydrate chains are thought to be important for understanding cell-microbe interactions; however, most of the previous studies focused on fungal pathogens, such as *Malassezia* and *Candida*. The cell-microbe interaction via carbohydrate chains of bacteria, including LAB, remains unclear. In this study, we performed lectin array analysis to examine the lectin binding patterns of carbohydrate chains on the cell membrane of LAB, including KW3110. Lectin array is an effective technique in studying the structural patterns of glycan^22,23^. It is a type of microarray using many lectins arranged on a substrate, each of which can bind to different carbohydrate chains, and is used for fast and high-throughput glycan profiling of samples. Our studies showed that KW3110 had a higher binding affinity to the lectins which recognized the carbohydrate chains comprised of α-mannose than other LAB. We then investigated which type of lectin receptor on the surface of RAW 264.7 cells had the potential to bind α-mannose. Lectin receptors, a large family of soluble transmembrane proteins, contain one or more carbohydrate-recognition domains to recognize sugar chains. Among the lectin receptors, Dectin-1 (gene symbol: *Clec7a,* type lectin domain family 7 member A) and Dectin-2 (gene symbol: *Clec4n,* type lectin domain family 4 member N) have a wide range of immune responses, including phagocytosis^24^. Both Dectin-1 and Dectin-2 are members of the C-type lectin family and are glycosylated type II transmembrane proteins with single carbohydrate recognition domains. Dectin-1 recognizes β-glucan with its carbohydrate recognition domains^25^, whereas Dectin-2 recognizes α-mannose^7^. Both receptors are important in the recognition and phagocytosis of microbes, and contribute to inducing cytokine and ROS production via the Syk-CARD9-NF-kB pathway^16,21^. We showed that KW3110 significantly increased the expression levels of *Clec4n* in RAW 264.7 cells when compared with NRIC1942 and LGG. The uptake amount of KW3110 by RAW 264.7 cells was significantly suppressed by knock-down of *Clec4n*. Furthermore, transcription analysis by RNA-seq showed that *Clec4n* was also the most up-regulated gene in the category of C-type lectin receptor signaling pathway genes in response to KW3110 stimulation. Whereas *Clec4n* gene expression was not up-regulated in response to stimulation by LGG or NRIC194. These results suggested that Dectin-2 in RAW 264.7 cells plays an important role in phagocytosis of KW3110.

Although previous reports showed that Dectin-2 mediates the phagocytosis of cancer cells by Kupffer cells and the phagocytosis of mycobacterium by dendritic cells (DC)^26,27^, to our knowledge, there are no reports that Dectin-2 is involved in the uptake of LAB. A previous study reported that the uptake of *L. reuteri* ATCC53608 was not affected when Dectin-2 was blocked in DC^28^. The results suggest that the involvement of Dectin-2 in phagocytosis depends on the LAB species and differences might be regulated by the structure of carbohydrate chains. Mincle is also reported to be a lectin receptor, recognizing α-mannose; a recent study reported that *Malassezia* is cooperatively recognized by Mincle and Dectin-2 through distinct ligands^29^. In this study, the uptake amount of KW3110 by si-Mincle cells did not significantly differ from si-control cells; suggesting that Mincle is not involved in at least phagocytosis of KW3110 by RAW 264.7 cells.

The engulfment of microbes by phagocytosis results in formation of an intracellular vacuole, termed the endosome. The fragment of LAB engulfed into the endosome is either collectively digested^30^ or transferred into the cell via a transporter. The POT family of proteins are peptide transporters^19^, comprised of four protein members: the peptide transporters PEPT1, PEPT2, and the di/tripeptides transporters PHT1, PHT2, which are expressed in various cells. *SLC15A1* (gene name of PEPT1) is abundantly expressed in the small intestine and *SLC15A2* (gene name of PEPT2) is expressed in various organs such as lung, brain and kidney, and various cell lines^31^. In vitro studies showed that PEPT1 is involved in facilitating the immune response^32^. PEPT2 is known to be a high-affinity and low-capacity transporter. Both *SLC15A4* (gene name of PHT1) and *SLC15A3* (gene name of PHT2) are expressed in various organs, including brain, placenta, lung, and thymus. PHT1 is reported to be associated with some diseases, including diabetes^33^. PHT2 is reported to play a role in antiviral innate immune responses^34^. A recent study also indicated that both PHT1 and PHT2 localized to endosomes and were responsible for the egress of fragments^35^. We found that *SLC15A4* and *SLC15A3* were expressed in RAW 264.7 cells, whereas *SLC15A1* and *SLC15A2* were not. PEPT1 and PEPT2 proteins were not expressed in CD11b+ macrophages^10^. These results suggest that PEPT1 and PEPT2 are not involved in phagocytosis in some macrophages, such as RAW264.7 and CD11b+ cells.

Interestingly, we demonstrated that only *SLC15A3* expression was increased in RAW 264.7 cells when stimulated by KW3110, whereas *SLC15A4* expression was not changed. We also showed that inhibition of *SLC15A3* resulted in suppression of IL-10 production in RAW 264.7 cells.

Bacterial MDP stimulated PHT2 in RAW cells which, in turn, facilitated the NOD2-dependent immune response^10^. *SLC15A3* is responsible for the egress of MDP^35^. IL-10 production after KW3110 treatment was abolished by the inhibition of NOD2 in bone marrow macrophages^2^. Taken together, it is suggested that phagocytosed KW3110 is digested in endosomes and the MDP of the phagocytosed KW3110 reaches the cytosol via PHT2 where it activates NOD2 signaling, leading to IL-10 production. Surprisingly, IL-10 production in KW3110-stimulated RAW 264.7 cells was increased with knock-down of *SLC15A4*. We also demonstrated that *SLC15A3* expression was significantly increased in si-PHT1 cells when compared to si-Control cells. Increased expression of *SLC15A4* may contribute to the activation of MDP transport, leading to IL-10 production (Supplemental Fig. 3b).

**Figure 7:**
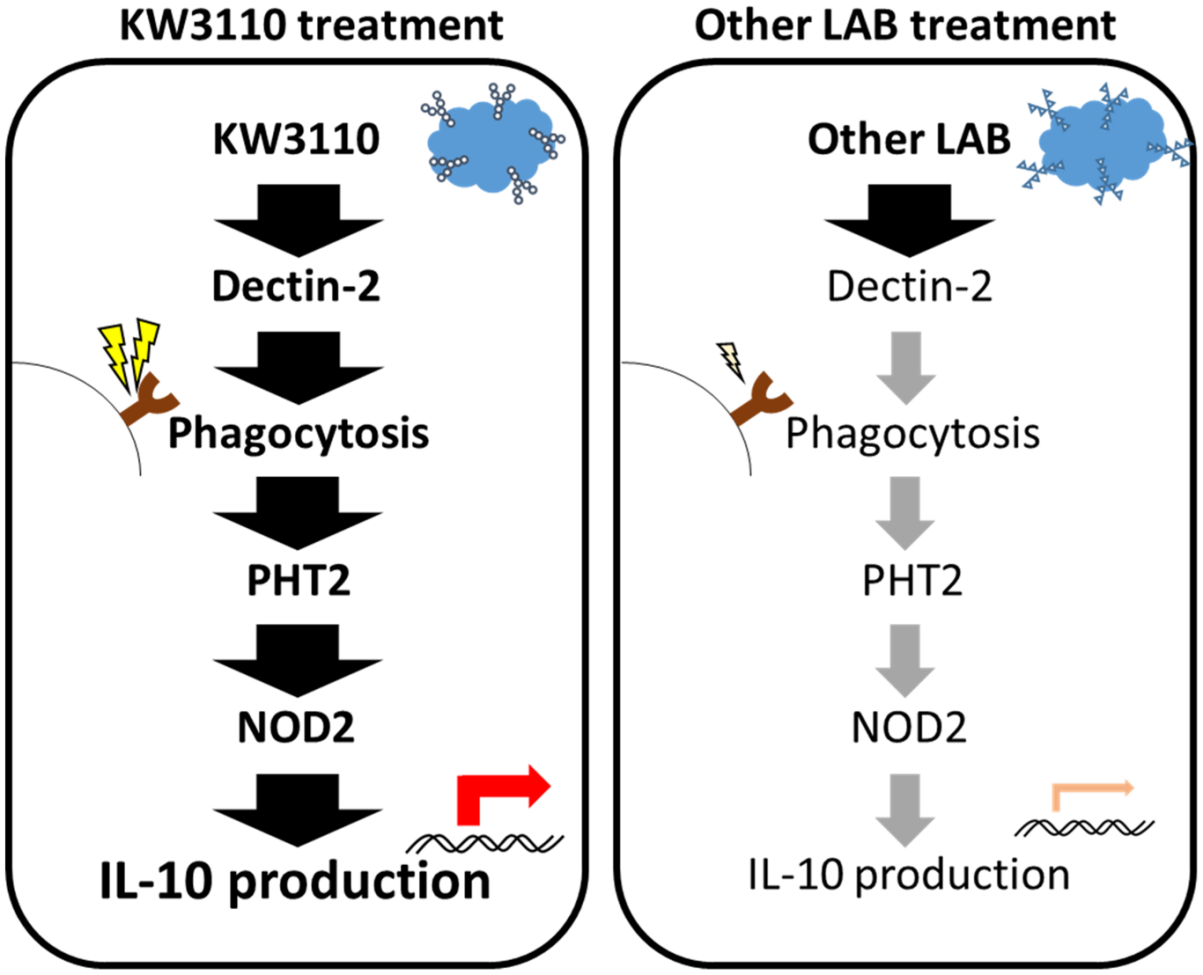
A signaling diagram of IL-10 production by KW3110 in RAW264.7 cells. KW3110 had higher binding affinities to lectins, which recognize the carbohydrate chains comprised of α-mannose, than other LAB. KW3110 is easily incorporated into RAW 264.7 cells. Dectin-2 expression in RAW 264.7 cells has a key role in phagocytosis of KW3110. After phagocytosis, the engulfed KW3110 might reach the cytosol via PHT2 and activate the IL-10 signaling pathway, including NOD2.

In conclusion, we showed that KW3110 is easily incorporated into RAW 264.7 cells, and leads to IL-10 production. Using si-RNA techniques, we revealed that Dectin-2 expression in RAW 264.7 cells has a key role in phagocytosis of KW3110. In addition, the expression levels of *SLC15A3* were high in KW3110 stimulated RAW 264.7 cells. After phagocytosis, the engulfed KW3110 may reach the cytosol via PHT2 and activate the IL-10 signaling pathway (Fig. 7).

## Methods

### LAB strains

KW3110 tested in this study were maintained at Koiwai Dairy Products Co., Ltd. (Saitama, Japan). NRIC1942 and LGG were purchased from collections held at the Culture Collection Center, Tokyo University of Agriculture (Tokyo, Japan). Cultures of LAB strains were grown at 30 °C or 37 °C for 48 h in MRS broth (Oxoid, Hampshire, UK) according to manufacturer’s instructions. Cultured LAB strains were washed twice with sterile distilled water, heat-killed at 70 °C, lyophilized, and suspended in PBS for in vitro studies.

### Cell culture and stimulation by heat-killed KW3110

RAW 264.7 murine macrophages were obtained from the American Type Culture Collection (Bethesda, MD, USA). Cells were cultured in DMEM supplemented with 10% FBS, streptomycin (100 μg/ml), and penicillin (100 units/ml) then incubated overnight at 37 °C and 5% CO_2._ For the experiments, RAW 264.7 cells were seeded and incubated overnight in 24-well plates (2.5 × 10^5^ cells/well), and then treated with heat-killed KW3110 (10 μg/ml) or other LAB (10 μg/ml) for 24 h in confluent state. RAW 264.7 cells were treated with heat-killed KW3110 at a concentration of 1.25, 2.5, 5, 10 μg/ml to verify concentration dependence.

### Reagents and antibodies

Cytochalasin D was purchased from Sigma (St. Louis, MO, USA). To block phagocytosis, Cytochalasin D was added 30 min prior to KW3110 stimulation. Gefitinib was obtained from Tocris Bioscience (Bristol, Avon, UK). Anti-mouse Dectin-2 antibodies were from MyBioSource (San Diego, CA, USA).

### ELISA

Cell culture supernatants were assayed for mouse IL-10 (BD Biosciences, San Jose, CA, USA), according to manufacturer’s instructions.

### RNA extraction and real-time PCR analysis

Total RNAs were extracted using an RNeasy Mini Kit (Qiagen, Venlo, Netherlands), following manufacturer’s recommendation for RNA preparation from bacterial cells, and reverse transcribed into cDNA using iScript cDNA synthesis kit (Bio-Rad Laboratories, Hercules, CA). The cDNA was used as a template in PCR reactions using TB Green premix Ex Taq (Takara Bio, Shiga, Japan). PCR was performed with specific primers listed in Table 1 using a LightCycler 480 (Roche Diagnostics, Tokyo, Japan). The PCR conditions were as follows: 2‑step cycling, 95 °C for 10 s hold; 45 cycles of 95 °C for 5 s and 60 °C for 20 s. Values were then normalized against the amount of glyceraldehyde-3-phosphate dehydrogenase (GAPDH) in each sample.

**Table 1:** Primer sequences used for quantitative real-time PCR.

Gene	Forward	Reverse
*GAPDH*	TGTGTCCGTCGTGGATCTGA	TTGCTGTTGAAGTCGCAGGAG
*CLEC7A*	AATCCTGTGCTTTGTGGTAG	GACTGAGAAAAACCTCCTGTAG
*Clec4n*	ATTTCATCACCCAGCAGC	AAAACATCATTC CA GCCCC
*Syk*	CTGGTTCCATGGCAACATCTC	TGGCCCTGATCAGGAATTTTC
*SLC15A1*	GCCGGACCAGATGCAGACGG	GCGGGTACACCACAGCGTCC
*SLC15A2*	ATGGTGCTGGCACTTGTTGTGT	AACGGTTCCTGAAGCGGTTGCA
*SLC15A4*	GCTGCCACCTGCATTACTACTTC	CGTACTTCACAGACACAATGAG
*SLC15A* 3	GCTGAAGCTTGCGTTCCAA	AACAGGTGGGCACTTTCAGAGT

### Transfection

RAW 264.7 cells were seeded into 12-well plates (3.0 × 10^5^ cells/well) and transfected with 100 nM small interfering RNAs (siRNA) using DharmaFECT 1 Transfection Reagent (Dharmacon, Lafayette, CO, USA) for 24 h before KW3110 treatment. siRNAs were all siGENOME SMARTpool siRNA (Dharmacon, Lafayette, CO, USA). Non-targeting siRNA (siNT, negative control), mouse *CLEC7A* siRNA (siDectin-1), mouse *CLEC4N* siRNA (siDectin-2), mouse *SYK* siRNA (siSyk), mouse *SLC15A4* siRNA (siPht-1), and mouse *SLC15A3* siRNA (siPht-2) were used in this study.

### Flow cytometric analysis

After pre-incubation of RAW 264.7 cells for 24 h, FITC-labeled LAB (10 μg/ml) were added. After 24 h, cells were washed with DMEM and resuspended in 0.2% trypan blue for 5 min to quench the fluorescence of non-ingested, but membrane-associated bacteria. The macrophages were suspended in 4% paraformaldehyde (Wako, Osaka, Japan) for flow cytometry analysis. Flow cytometric analysis was performed on a FACS Canto II (BD Biosciences, San Jose, CA, USA) and analyzed using FlowJo software. Cell populations were gated by the software program in the scatter diagram (forward-scattered light versus side-scattered light). FITC-positive population was defined as over 10^3^ of intensity. The uptake quantity was determined as Cell Number × MFI.

### Microscopic analysis of phagocytosis

After pre-incubation of RAW 264.7 cells for 24 h, FITC-labeled LAB (10 μg/ml) were added. After 24 h, cells were washed with DMEM and suspended in 0.2% trypan blue for 5 min; cells were then washed in and resuspended in PBS. RAW 264.7 cells were observed by confocal laser scanning microscope FV1000 (OLYMPUS, Tokyo, Japan).

### Lectin array

The lectin microarray was performed using RayBio Lectin Array 95 (RayBiotech, Inc. Norcross, GA). All LAB species were dissolved at a concentration of 0.2 mg/ml in PBS and used as samples. After their addition to labeling reagent for 1 h, the samples were dialyzed and centrifuged at 1000 rpm for 5 min at 4 °C. Hybridizations were performed on the supernatant according to the manufacturer’s protocol.

### Transmission electron microscopy

RAW 264.7 cells were grown for 24 h on 35 mm dishes (5.0 × 10^5^ cells). Cells were washed with PBS and exposed to 1 μg/m1 KW3110 in DMEM for 24 h. After fixation, each cell sample was consigned to Tokai Electron Microscopy Inc. (Aichi, Japan). Cells were washed with PBS buffer and postfixed in 2% osmium tetroxide for 2 h at 4 °C and embedded in resin (Quetol-812) after dehydration in ascending grades of ethanol. Ultrathin sections, 70 nm, were cut and stained with alkaline and lead citrate uranyl acetate. The sections were examined under a TEM (JEM-1400Plus, JEOL Ltd., Tokyo, Japan).

### RNA sequencing

Briefly, total RNAs were extracted from RAW 264.7 cells using RNeasy mini columns (Qiagen, Venlo, Netherlands). Library preparation and sequencing were consigned to Takara Bio Inc. Sequencing of each library was performed on a NovaSeq 6000 (illumina, San Diego, California, USA). Sequencing was performed to generate 150 bp paired-end reads.

### RNA-seq data analysis

All RNA‐seq data are deposited in the Gene Expression Omnibus database at the National Center for Biotechnology Information. Analysis of the RNA-seq data was consigned to Takara Bio Inc. Preprocessing of the RNA‐seq data was completed using Genedata Profiler Genome. All samples passed quality control criteria. Reads were mapped to the GRCm38 mouse genome using STAR. The category classifications of genes were referred to KEGG^36–38^.

### Statistical analysis

Statistical differences were evaluated using the Student's *t*-test for comparison of two groups or analysis of variance (ANOVA) followed by Dunnett’s or Tukey’s test for multiple comparisons. *p*-value < 0.05 was considered significant.

## Supplementary Information

Supplementary Information.

## Data Availability

The datasets analyzed during the current study are available from the corresponding authors on reasonable request.
