# Supplementary material for: Dectin-2 mediates phagocytosis of Lactobacillus paracasei KW3110 and IL-10 production by macrophages
Source: Sci Rep. 2021 Sep 6;11:17737. doi: 10.1038/s41598-021-97087-9 (PMC8421511; doi:10.1038/s41598-021-97087-9)
Supplement: Supplementary file 1 — Supplementary Information. [file 41598_2021_97087_MOESM1_ESM.docx]

**Title**

**Dectin-2 mediates phagocytosis of *Lactobacillus paracasei* KW3110 and IL-10 production by macrophages**

**Author**

Mia Yoshikawa ^1^, Sayuri Yamada ^1^, Miho Sugamata ^1^, Osamu Kanauchi ^1^, Yuji Morita ^1^

^1^; KIRIN Central Research Institute, Kirin Holdings Company, Ltd.,

**
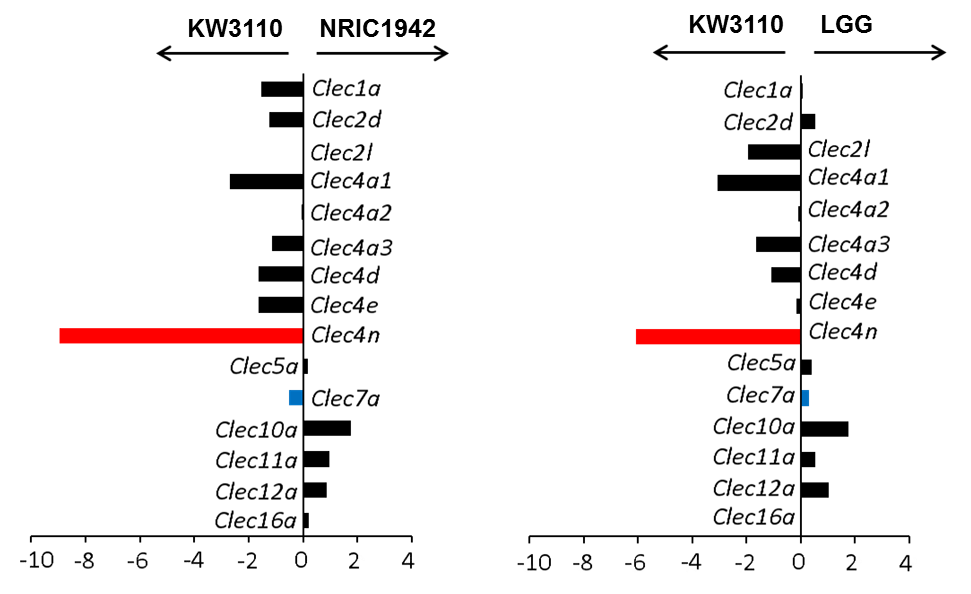
**

**Supplemental Figure 1. Dectin-2 in RAW 264.7 cells is involved in incorporation of KW3110 via reorganization of mannose.**

Differential gene expression levels of C-type lectin domain in KW3110-stimulated versus other *Lactobacilli*- stimulated cells. Relative gene expression levels are compared between RAW cells stimulated by KW3110 vs NRIC1942 or LGG. Relative gene expression levels of *Clec4n* and *Clec7a* are represented by red and blue bars.

**
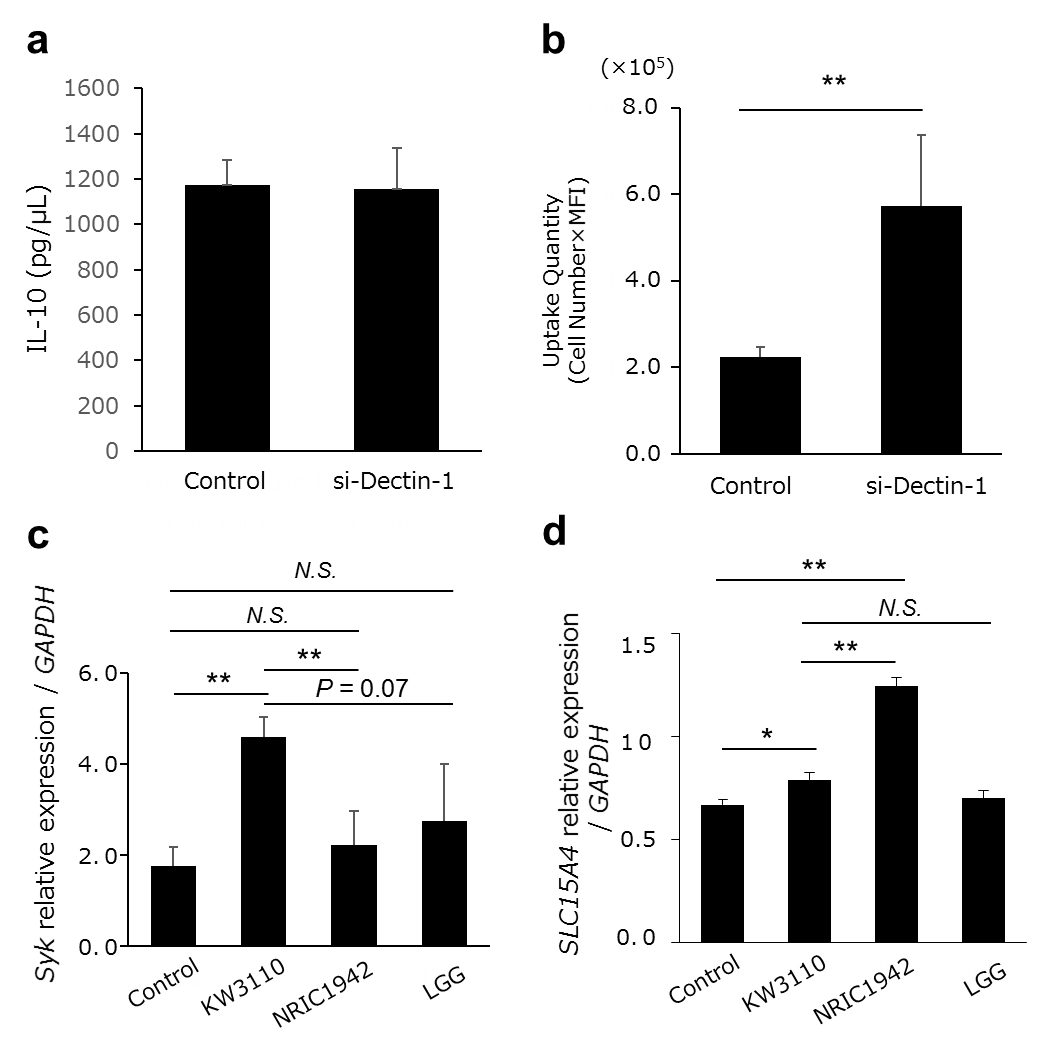
**

**Supplemental Figure 2. Dectin-1 is not involved in IL-10 production.**

[a, b]

Dectin-1 knockout RAW 264.7 cells were treated with KW3110 for 24 h. [a] IL-10 concentration in the supernatants was measured by ELISA. [b] The amount of incorporated KW3110 was measured by flow cytometry.

[c]

*Syk* expression in RAW 264.7 cells stimulated by *Lactobacilli*. The relative expression levels of the gene normalized to GAPDH.

[d]

*SLC15A4* expression in RAW 264.7 cells stimulated by *Lactobacilli*. The relative expression levels of the gene normalized to GAPDH.

Significant differences were compared to the control group, ***p* < 0.01. Data are the means ± SD. Data are representative of at least three independent experiments.

**
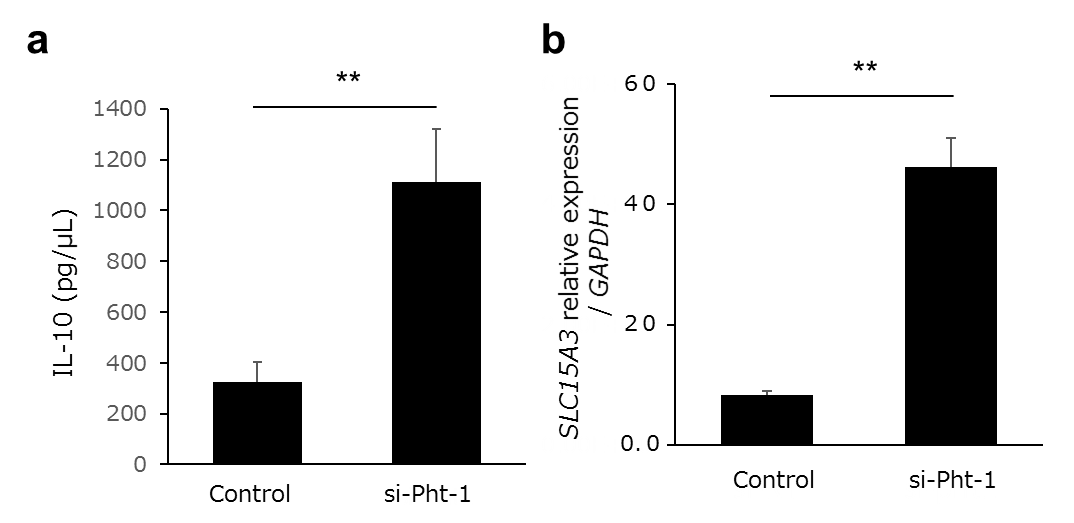
**

**Supplemental Figure 3. Inhibition of Pht1 increased IL-10 production.**

[a]

Pht1 knockout RAW 264.7 cells were treated with KW3110 for 24 h. IL-10 concentration in the supernatants was measured by ELISA.

[b]

*SLC15A3* expression in si-Pht1 cells stimulated by *Lactobacilli*. The relative expression levels of the gene normalized to GAPDH.

Significant differences were compared to the control group, ***p* < 0.01. Data are the means ± SD. Data are representative of at least three independent experiments.
